# Analysis of critical report notification from musculoskeletal radiology in a tertiary academic medical institution with a regional trauma center

**DOI:** 10.1371/journal.pone.0262511

**Published:** 2022-01-13

**Authors:** Tae Ran Ahn, Yu Mi Jeong, So Hyun Park, Ji Young Jeon, Sheen-Woo Lee, Young Sup Shim

**Affiliations:** Department of Radiology, Gil Medical Center, Gachon University College of Medicine, Incheon, Republic of Korea; Medical College of Wisconsin, UNITED STATES

## Abstract

**Purpose:**

We aimed to analyze the prevalence, causes, and clinical settings of 4-year critical radiologic reports (CRRs) notified from the musculoskeletal section of the radiology department. Then, we investigated the communication outcomes.

**Methods:**

This study was approved by our institutional review board. We retrospectively included 175 musculoskeletal CRRs from our database between January 2017 and December 2020. The CRRs were analyzed by two musculoskeletal radiologists, who categorized the CRRs by clinical setting (emergency department(ED) patient, outpatient, and inpatient), body part, type of image modality, reason for CRR, incidental lesion, and clinical outcome. The clinical outcome was retrieved from the electronic medical records.

**Results:**

The 175 musculoskeletal CRRs accounted for 5.4% of the CRRs (*n* = 3217) available in the study period. Most CRRs (94.9%, 166/175) corresponded to the musculoskeletal system, while the remaining ones (5.1%, 9/175) corresponded to the non-musculoskeletal system. In addition, the spine, extremities, and thoracic cage accounted for 52.6%, 40.6%, and 1.7% of the musculoskeletal CRRs, respectively. Moreover, most patients presented to the ED (50.3%, 88/175), followed by inpatients (30.9%, 54/175), and outpatients (18.9%, 33/175). The CRR reasons included missed fracture (54.3%), suspected malignancy (16%), clinical emergency (10.3%), unexpected infection/inflammation (11.4%), and others (8%). Furthermore, 11 (6.3%) incidental lesions were not related to the primary imaging purpose. Referring clinicians actively acknowledged 80% of the CRRs. The loss to follow-up action was the highest in the ED patients (35.2%, 31/88; *p* < 0.001), being significantly higher than that in outpatients (6.1%, 2/33) and inpatients (3.7%, 2/54).

**Conclusion:**

Missed fractures were the most common cause of musculoskeletal CRRs. ED showed prevalence in musculoskeletal CRRs and reflected the highest loss to follow-up action. ED physicians should pay more attention to CRRs to enhance patient care.

## Introduction

Medical imaging comprises an examination, a verified report, and the report communication [1]. Currently, routine official radiologic reports are documented through the Picture Archive and Communication System (PACS). However, critical or unexpected imaging findings with clinical significance may require timely non-routine communication. Optimal communication of critical radiologic reports (CRRs) has become more prevalent, especially after the American College of Radiology released standard guidelines for non-routine communication [2]. The detailed situations of non-routine communications by ACR guideline are as follows [2]: (i) findings that suggest a need for immediate or urgent intervention, (ii) findings that are discrepant with a preceding report of the same exam and where failure to act may adversely affect patient health, (iii) findings that the interpreting radiologist reasonably believes are significant and unexpected, may have a reasonable probability of impacting the patient’s health and may not require immediate attention but, if not acted on, may worsen over time and likely result in an adverse patient outcome.

Radiologic reports with clinical significance or requiring immediate action should be promptly informed to the referring physician to ensure the continuity of patient treatment. The Joint Commission has mandated compliance with the communication of critical test results among caregivers on a timely basis as an important part of the National Patient Safety Goals [3].

Non-routine communication varies depending on the institution, and many medical centers have implemented electronic systems for non-routine communication [4–6]. Thus, radiologists can timely alert the referring physician and their team by sending a text message related to CRRs using the notifications embedded in PACS. Despite the improving documentation of CRR non-routine communication [7, 8], to our knowledge, its analysis in specific subspecialties has not been addressed. In particular, musculoskeletal CRRs may be beneficial for patients with potentially missing risk management, highlighting the importance of their analysis.

In this study, we analyzed the prevalence, reasons, clinical settings, and follow-up rate related to CRRs in the musculoskeletal section and identified the impact of electronic notification systems and communication outcomes.

## Materials and methods

### Study design

This retrospective study was approved by the Gil Medical Center institutional review board and the requirement for informed consent was waived given the retrospective nature of this study.

### Patients

**Fig 1:**
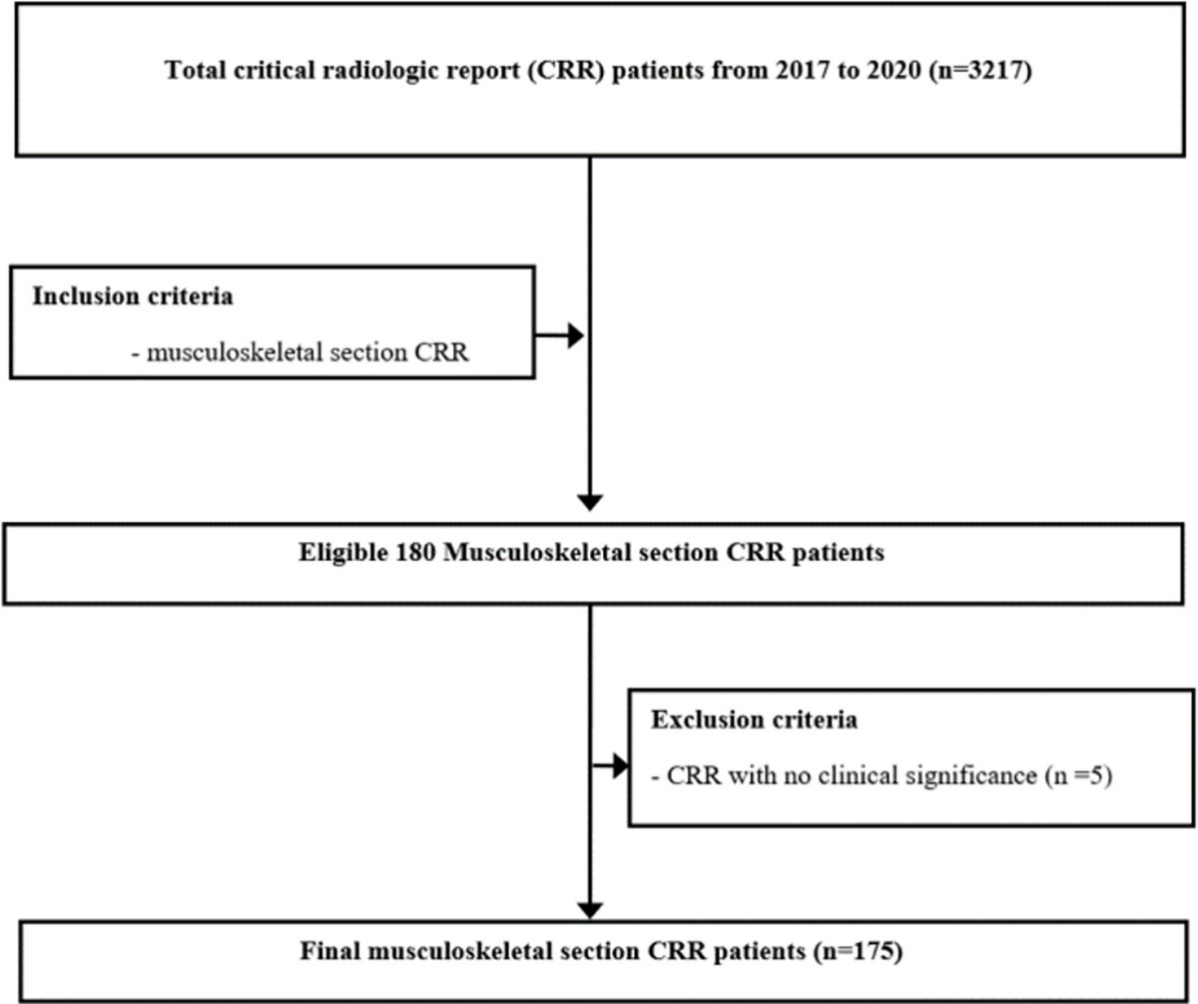
Flowchart of CRR selection.

Our institution is a tertiary-referral academic medical institution with 1700 beds and a national regional trauma center. An electronic alert notification system was embedded in PACS for CRRs in 2015. In total, 3217 consecutive CRRs were retrieved from the electronic medical record (EMR) system (BESTCare 2.0, Korea) from January 2017 to December 2020. Metadata including patient identification, patient age, referring department, and study name were automatically extracted during database search, obtaining 180 CRRs from the musculoskeletal section of the radiology department. As 5 cases showed no clinical significance (i.e., small disc herniation, mistake, or error of radiologist), we used 175 musculoskeletal CRRs for analysis (**Fig 1**).

### CRRs

We defined musculoskeletal CRR as a significant finding detected in musculoskeletal imaging studies to primarily intend to evaluate the musculoskeletal system including spine, extremities, and pelvic bone, finally read by musculoskeletal attending radiologists. The CRR information about each alert notification (i.e., examination name, sending time of text message, and physician receiving the notification) was documented in the corresponding EMRs. During the study period of 4 years, five radiologists worked in the musculoskeletal section, and they reported CRRs for emergency findings that needed urgent management or for clinically important findings (e.g., trauma, tumor, infection) that were considered unrecognized by the referring physician.

### CRR analysis

Two musculoskeletal radiologists with 12 and 4 years of experience reviewed the EMRs and magnetic resonance imaging (MRI) reports with image findings of each musculoskeletal CRR case. We categorized the data by clinical setting (emergency department(ED) patient, inpatient, outpatient), body part (musculoskeletal, non-musculoskeletal), imaging modality (MRI, computed tomography(CT), ultrasound, X-ray), reason for CRR (e.g., clinical emergency with need of immediate management due to pseudoaneurysm, necrotizing fasciitis, or cord compression; missed fracture; suspected malignancy; unexpected infection/inflammation), and incidental lesion or not.

### Follow-up after CRR

The CRR was considered as actively acknowledged when clinical notes were available about the radiology result notification described by physicians in the EMR or when additional management (e.g., additional imaging, biopsy, consultation to another department, and treatment) was performed after the CRR notification. The clinical outcomes of each CRR were obtained from the corresponding EMRs and classified as surgical treatment/intervention, medical treatment, conservative management, and telephonic notification to patient. On the other hand, loss to follow-up action was considered for unacknowledged CRRs.

### Statistical analysis

Descriptive statistics were compared among the causes, clinical settings, and follow-up cases using chi-squared tests implemented in SPSS (version 26.0; IBM, Armonk, NY, USA). Statistical significance was set at *p* < 0.05.

## Results

The characteristics of the 175 study subjects (101 males, 74 females) are listed in **Table 1**. The age range of the subjects was 7–84 years with mean of 54.8 years. In the clinical setting, most patients were presented to the ED (50.3%, 88/175), followed by inpatients (30.9%, 54/175) and outpatients (18.9%, 33/175). Radiography (41.7%, 73/175) was the most frequent imaging modality followed by MRI (34.3%, 60/175), CT (22.9%, 40/175), and ultrasound (1.1%, 2/175).

**Table 1:** Characteristics of the study subjects.

	Value
**Total number of musculoskeletal CRR**	175
**Mean age (year)**	54.8
**Clinical setting**	
1. ED patient	88 (50.3)
2. Inpatient	54 (30.9)
3. Outpatient	33 (18. 9)
**Body part**	
1. Musculoskeletal system*	**166 (94.9)**
*(1) Spine*	*92 (52*.*6)*
• *Cervical*	*40 (22*.*9)*
• *Thoracic*	*17 (9*.*7)*
• *Lumbar*	*35 (20)*
*(2) Extremity*	*74 (40*.*6)*
• *Upper extremity*	*28 (16)*
• *Lower extremity*	*43 (24*.*6)*
*(3) Thoracic cage*	*3 (1*.*7)*
2. Non-musculoskeletal system **	**9 (5.1)**
**Types of image modality**	
1. X-ray	73 (41.7)
2. MRI	60 (34.3)
3. CT	40 (22.9)
4. Ultrasound	2 (1.1)

Note. Data are presented as number (%), unless indicated otherwise.

*Musculoskeletal system includes muscles, tendons, ligaments, nerves, discs, and blood vessels.

** Non-musculoskeletal system includes abdomen, chest, and brain.

The CRRs from the musculoskeletal imaging accounted for 5.4% (*n* = 175) of the available CRRs (*n* = 3217). Most musculoskeletal CRRs (94.9%, 166/175) corresponded to the musculoskeletal system, and the remaining CRRs (5.1%, 9/175) corresponded to the non-musculoskeletal system (i.e., abdomen, chest, brain). In the musculoskeletal system, the spine cases (52.6%, 92/166) outnumbered cases in the extremities (40.6%, 74/166).

**Fig 2:**
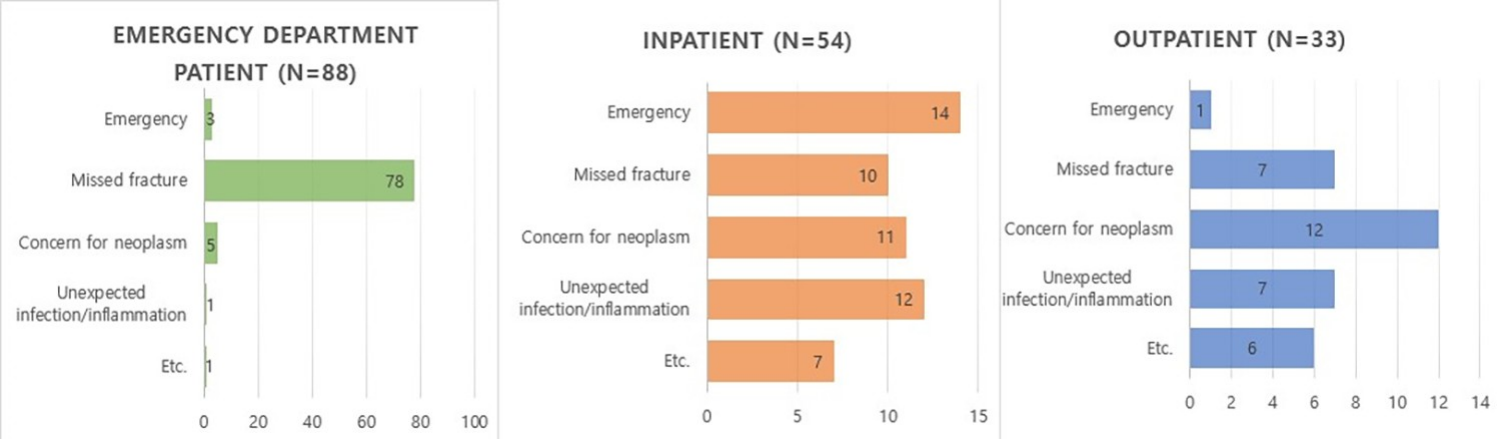
Reasons for CRR.

**Fig 3:**
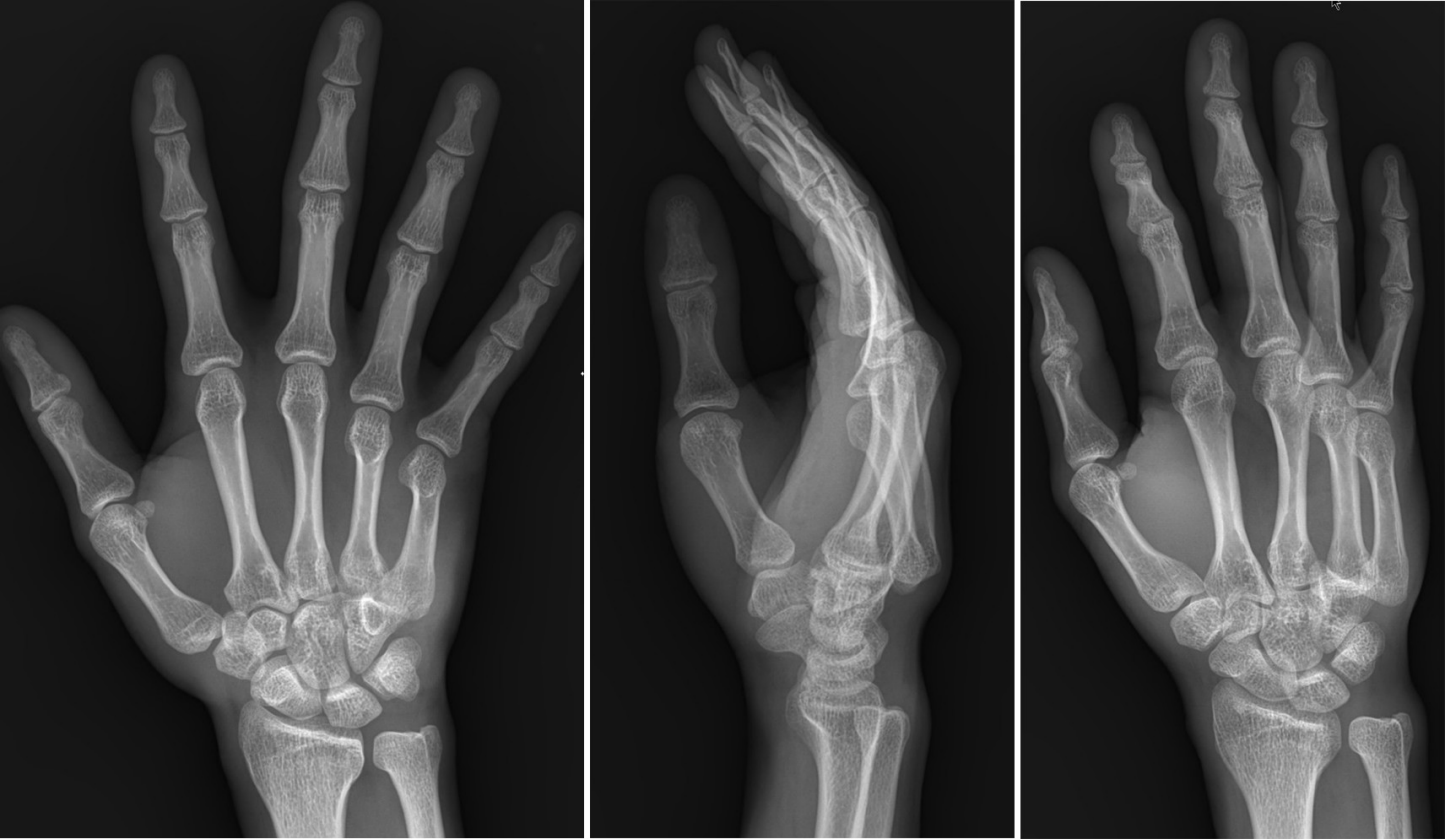
Dorsal dislocation of fourth and fifth carpometacarpal joint (ED patient, missed fracture, telephone call). A 28-year-old man visited the ED because of hand pain after falling. Hand X-ray anteroposterior view shows no significant abnormality (A), but lateral (B) and oblique (C) views clearly show dorsal dislocation of the fourth and fifth metacarpophalangeal joint. The ED informed the patient about this lesion via telephone after obtaining the CRR.

**Fig 4:**
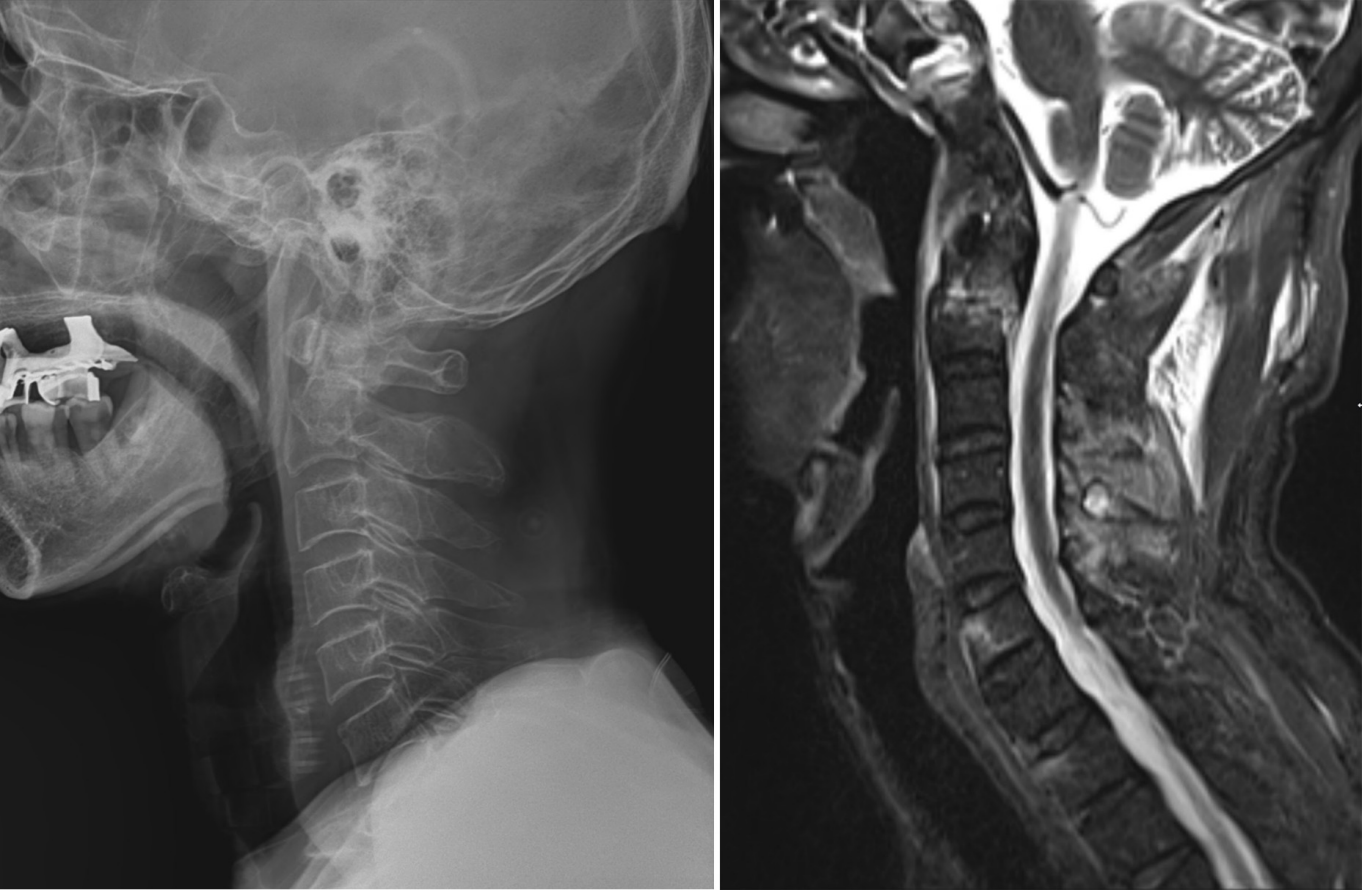
Odontoid process fracture (ED patient, missed fracture, conservative treatment). A 62-year-old man visited the emergency room because of neck pain caused by a fall while climbing a mountain. The C-spine X-ray lateral view shows a transverse fracture line across the odontoid process (A). On sagittal fat-suppressed T2-weighted MRI (B), transverse fracture of odontoid process is again noted. The patient underwent conservative treatment wearing a halo vest after obtaining the CRR.

**Fig 5:**
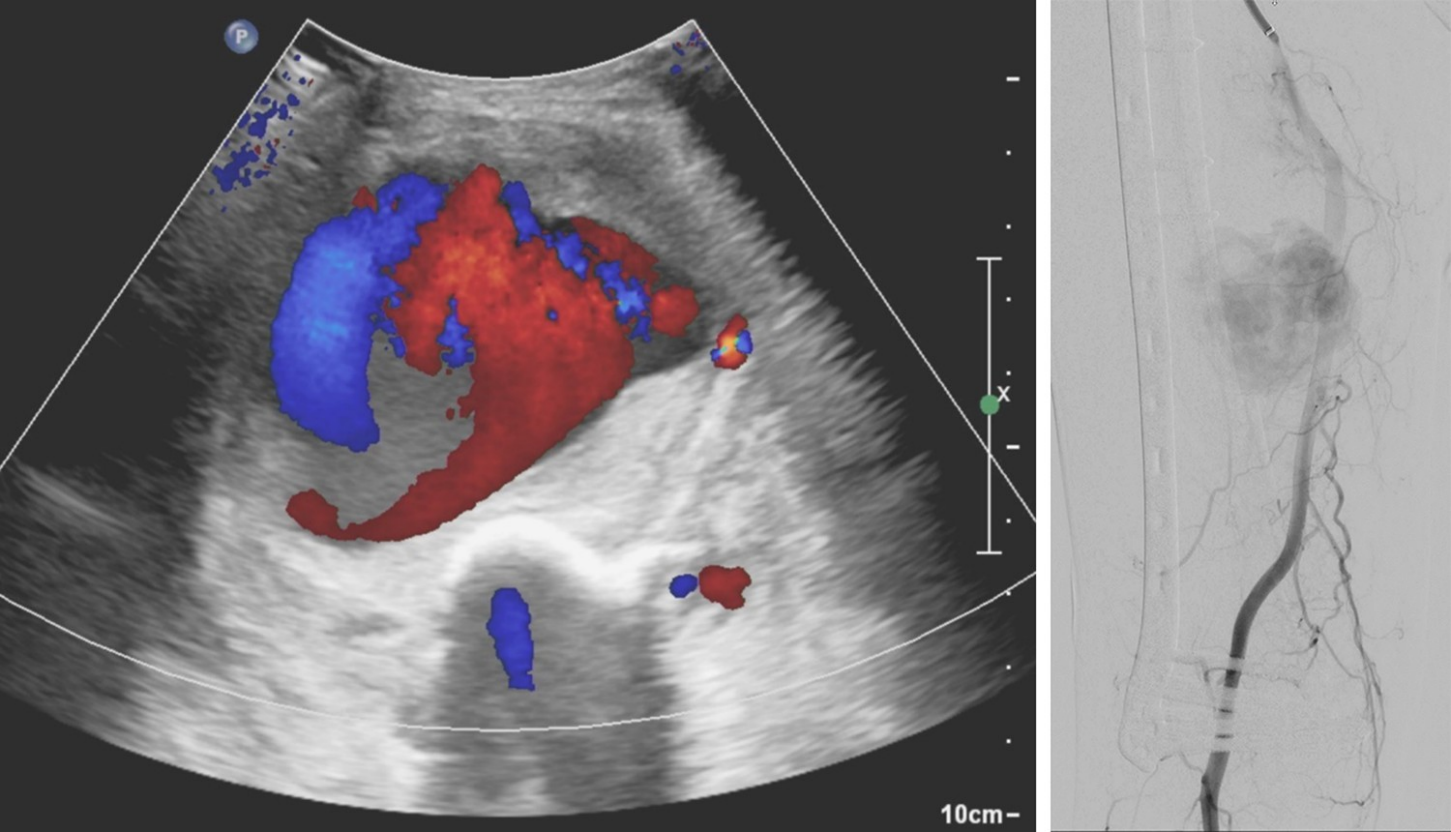
Pseudoaneurysm of superficial femoral artery (outpatient, emergency, interventional treatment). A 69-year-old man with a history of open reduction and internal fixation of the femur for a fracture that occurred 1 month before this examination complained of a palpable mass on his thigh. The characteristic yin-yang sign is noted on Doppler ultrasonography (A). Emergent femoral angiography shows a large pseudoaneurysm (B). The patient was treated with an endovascular stent graft.

**Table 2** and **Fig 2** show the reasons for the CRRs. Missed fractures (54.3%, 95/175; **Figs 3** and **4**) were the most common reasons followed by suspected malignancy (16.0%, 28/175), unexpected infection/inflammation (11.4%, 20/175), clinical emergency (10.3%, 18/175; **Fig 5**), and others (e.g., hardware complication, myelopathy, foreign body, dural arteriovenous fistula; 8.0%, 14/175). However, there were significant differences in the reasons according to the clinical setting. Missed fractures were significantly more numerous in the ED than in other clinical settings (*p* < 0.001). The most common reason per clinical setting was missed fractures (78/88) in ED patients, clinical emergency (14/54) in inpatients, and suspected malignancy (12/33) in outpatients.

**Table 2:** The reason for critical report notification.

Reason for CRR	Cases No.
1. Clinical emergency (*i*.*e*. active bleeding, necrotizing fasciitis, cord compression,)	18 (10.3)
2. Missed Fracture (refer to Table 2 for detail)	95 (54.3)
3. Concern for malignancy	28 (16)
4. Unexpected infection/Inflammation	20 (11.4)
5. Others (*i*.*e*. hardware complication., myelopathy, foreign body, dural AVF)	14 (8)

Note. Data are presented as number (%), unless indicated otherwise.

**Table 3** lists the missed fractures according to the body part. Most fractures were missed in the spine (45.3%, 43/95) followed by lower extremity (26.3%, 25/95), upper extremity (23.2%, 22/95), and thoracic cage (3.2%, 3/95). The C-spine (65.1%, 28/43) was the most common site of a missed spinal fracture.

**Table 3:** Distribution of missed fractures.

	Location	*n (95)*
Axial skeleton	Spine	43
	• *Cervical*	*28*
	• *Thoracic*	*6*
	• *Lumbar*	*9*
	Sacrum	2
Upper extremity	Shoulder	3
	Elbow	8
	Wrist and hand	11
Lower extremity	Hip	9
	Knee	7
	Lower leg	1
	Foot & Ankle	8
Thoracic cage	Rib	2
	Sternum	1

Note. Data are presented as number (%), unless indicated otherwise.

**Fig 6:**
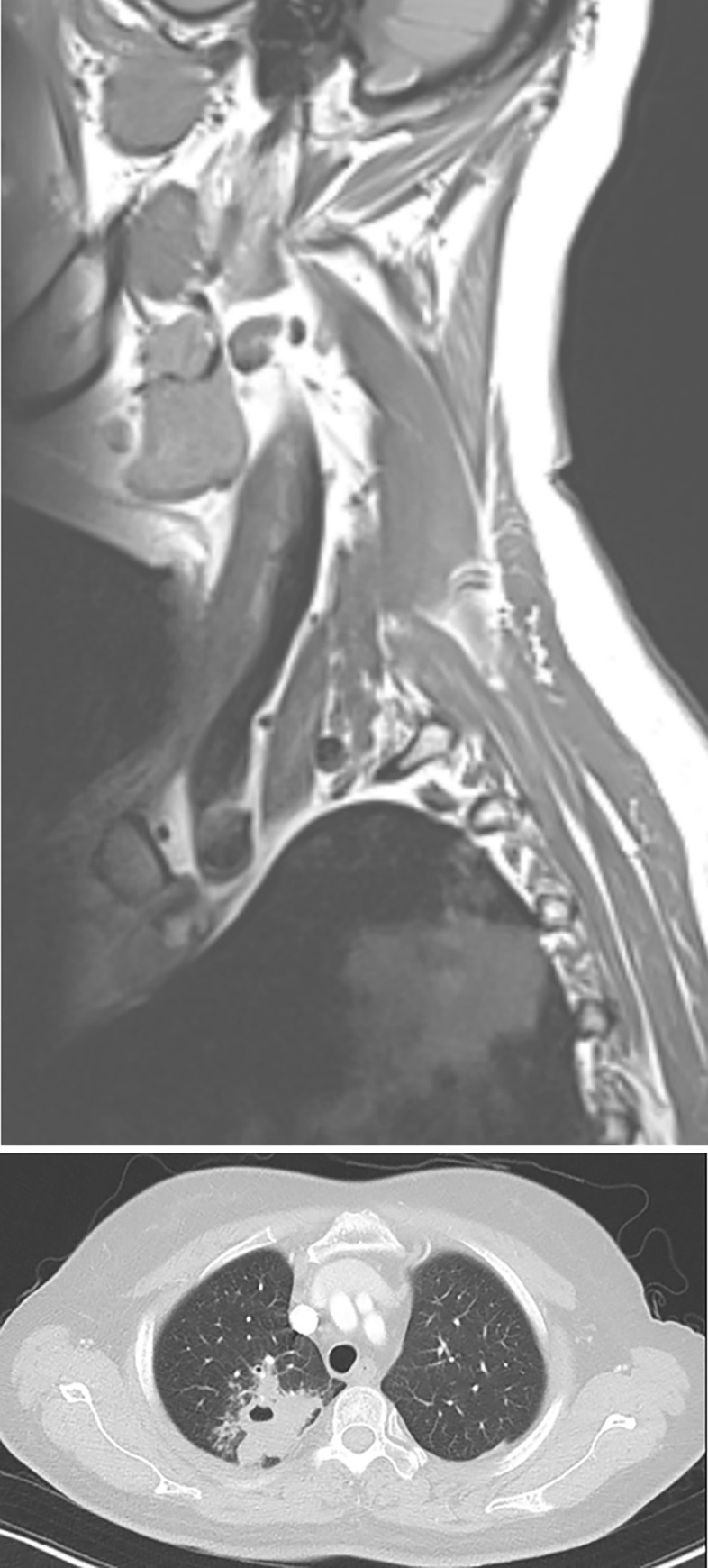
Pulmonary tuberculosis (outpatient, incidental lesion, medical treatment). A 60-year-old woman visited our outpatient clinic with bilateral numbness. C-spine MRI (A) allowed to determine a herniated disc at C6-7 (not shown) and incidentally noted consolidation in the right upper lung. Chest CT after CRR shows a large cavitary lesion in the right upper lobe (B). The patient underwent the QuantiFERON test, which was positive for tuberculosis infection and was treated with four-drug therapy.

From the cases, 11 (6.3%) incidental lesions in the brain, lung, retroperitoneum, and musculoskeletal system were detected from spine MRI, spine CT, and shoulder CT (**Table 4** and **Fig 6**).

**Table 4:** Clinically unexpected incidental lesions.

Age/Sex	Location	Specific site	Final diagnosis	Radiologic examination
28/F	Brain	Pituitary gland	Macroadenoma	Cervical spine MRI
47/M	Brain	Cerebellum	Metastasis	Cervical spine MRI
71/M	Brain	Cerebrum	Metastasis	Cervical spine MRI
47/M	Thorax	Lung	Tuberculosis	Shoulder CT
60/F	Thorax	Lung	Tuberculosis	Cervical spine MRI
31/M	Thorax	Lung	-*	Thoracic spine CT
81/M	Hip & Pelvis	Femoral head	Avascular necrosis	Lumbar spine MRI
26/M	Hip & Pelvis	Sacroiliac joint	Sacroiliitis	Lumbar spine MRI
38/F	Retroperitoneal space	Kidney	Angiomyolipoma	Lumbar spine MRI
57/F	Retroperitoneal space	Paraaortic space	Indeterminate lymph node	Lumbar spine CT
62/F	Retroperitoneal space	Paraaortic space	Metastatic lymph node from cervical cancer	Lumbar spine MRI

*Follow up loss.

**Fig 7:**
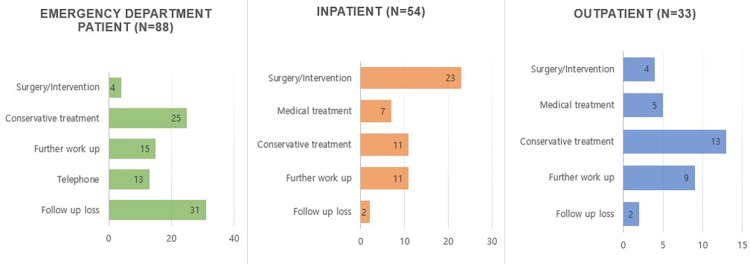
Management and follow-up after CRR.

**Fig 7** shows the management and loss to follow-up action after CRR according to the clinical setting. The active acknowledgement rate of CRR was 80% (140/175), with loss to follow-up action occurring for the remaining 20% (35/175) of cases (Table 5 (a)). The loss to follow-up action was higher in the ED patients (35.2%, 31/88) than in the inpatients (3.7%, 2/54) and outpatients (6.1%, 2/33) (*p* < 0.001). All the 13 telephonic notifications were identified only in the ED patients. The details about the cases of loss to follow-up action are shown in Table 5 (b).

**Table 5:** Clinical outcome of musculoskeletal CRR (a) and follow-up loss (b) cases characteristics.

**(a)**					
**Clinical outcome**				Patient No.	
1. Actively acknowledgement				**140 (80)**	
*(1) Surgical treatment/intervention*				*31*	
*(2) Medical treatment*				*12*	
*(3) Conservative treatment*				*49*	
*(4) Further work up ((lab test*, *image exam*, *PET CT*, *biopsy)*				*35*	
*(5) Telephone notification to patient*				*13*	
2. Loss of follow-up				**35 (20)**	
**(b)**					
Follow-up loss cases: Reason of CRR	Total patients (n = 35)	ED patient (n = 31)	Inpatient (n = 1)		Outpatient (n = 3)
1. Clinical emergency	0	0	0		0
2. Missed fracture	31	31	0		0
3. Concern for malignancy	1	0	0		1
4. Unexpected infection/inflammation	1	0	0		1
5. Others*	2	0	1		1

Note. Data are presented as number (%), unless indicated otherwise.

*Include large herniated disc on cervical trauma CT, Dural AVF, and myelopathy on MRI.

## Discussion

We analyzed the usage of an alert notification system for CRRs in the musculoskeletal section. The analysis included prevalence, causes, clinical settings, and whether the appropriate follow-up action was taken after the CRR. The results revealed that musculoskeletal CRRs accounted for 5.4% of the total CRRs. Missed fractures were the most common cause of musculoskeletal CRRs, with ED patients showing most of these cases. The active acknowledgement rate of musculoskeletal CRRs was 80%, whereas follow-up action was lost in 20% of CRRs. However, the results differed depending on the clinical setting (ED patients, inpatients, or outpatients). The rate of loss to follow-up action was the highest in the ED.

Few studies have reported that musculoskeletal imaging shows a relatively low prevalence of CRRs (4.5% [8], 19.3% [9]), requiring communication beyond the formal report. Therefore, scarce research is available on non-routine communication regarding musculoskeletal imaging. Consistent with the aforementioned studies [8, 9], we found that musculoskeletal CRRs had a low prevalence, accounting for only 5.4% of the CRRs. This low incidence may be explained by the musculoskeletal system having a low proportion of alert categories including urgent life-threatening results and life-threatening findings [8], compared with other body parts.

Missed fractures were the most common cause of musculoskeletal CRRs in our study, with the highest prevalence in the ED. Missed fractures represent up to 80% of diagnostic errors in the ED [10], and it frequently lead to legal problems in medicine [11]. Given the radiology workforce shortage and emergent clinical situations, physicians in the ED should often make management decisions before radiologic reports become available, especially considering plain radiographs [12, 13]. In our study, the cervical spine was the most common location of missed fractures, showing consistency with various studies that have reported that plain radiography may lead to miss more than 50% of cervical spine fractures for reasons including inadequate cervical spine series (e.g., lateral view only, non-visualization of C7-T1) and misreading of plain radiographies with or without adequate standard series [10, 14, 15]. In the extremities, the wrist and hand have been reported among the most common locations of missed fractures on plain radiographies [16, 17]. Wei et al. [16] showed that the wrist is the most frequent location for missed fractures, and the foot is the most frequent location expressed as percentage in the same location for extremity missed fractures. In addition, Guly [11] reported that fractures in the wrist are the second most frequently missed. In the pediatric population, Mounts et al. [17] reported that the most frequent missed fractures occur in the hand phalanges, followed by the metatarsal bone, distal radius, and tibia. Similarly, we found that the wrist and hand are the most frequent locations for missed fractures in the extremities.

Several infectious, inflammatory, and vascular emergencies also affect the musculoskeletal system [18–20], whereas medical emergencies in the musculoskeletal system are commonly secondary to trauma. However, clinical emergencies were rare in the musculoskeletal CRRs (10.3%, 18/175) considered in our study. In emergencies, such as open fracture, active bleeding, necrotizing fasciitis, and cord compression, rapid management could be performed ahead of formal radiologic reports due to the definite clinical findings, possibly decreasing the rate of CRRs in clinical practice.

We also found that 6.3% (11/175) of incidental lesions in the brain, lung, retroperitoneum, and bone and joint were detected on spine MRI, CT, and shoulder CT. In approximately half of these cases, CRRs were generated by suspected malignancy. To prevent unexpected radiologic findings deriving in mortality or morbidity, CRRs due to incidentally suspected malignancy are important for patient safety. With the development of imaging techniques, the diagnostic performance has improved, and the frequency of incidental findings that are unrelated to the primary purpose of examination has increased [21]. In whole-body MRI of the general adult population, up to 36% of potentially relevant incidental findings have been reported [22]. As musculoskeletal imaging covers various body parts, many unexpected incidental lesions may be revealed [23–25]. One meta-analysis provided a mean frequency of incidental findings in imaging diagnostic tests of 23.6%, mean frequency of clinical follow-up of 64.5%, and mean frequency of clinical confirmation of 45.6% [26]. Clinicians who order imaging studies in the trauma setting usually pay attention to detect bone or soft tissue injuries. This study can help clinicians to learn specific missed traumatic and non-traumatic lesions in musculoskeletal imaging, which will improve patient care as well as reduce the follow up loss rate.

We found a 20% loss to follow-up action, possibly due to ineffective communication between the radiologists and referring physicians or to the patient’s disagreement to subsequent work-up or treatment. Roy et al. [27] reported that when patients were discharged from hospitals with pending examination results, physician unawareness of actionable results could reach up to 62%, potentially leading to adverse outcomes. Non-routine communication of clinically significant findings may ensure the review of reports by a clinician [6]. Sahraian et al. [28] assessed the utilization of reports and images in musculoskeletal radiology, with only 0.8% of referring physicians reviewing images without radiologic reports. However, in the ED, viewing images before the formal radiologic reports was much more frequent, accounting for 9.7% of the cases [28]. Consistently, the rate of active acknowledgement was significantly lower in ED patients (64.8%, 57/88; *p* < 0.001) than in inpatients or outpatients. Therefore, clinicians being acquainted with missed lesions during emergencies can help to reduce the loss to follow-up action rate. Telephonic follow-up is being increasingly focused on patient management, particularly in the ED [29, 30]. In our study, 7.2% of the CRRs initially undetected by the clinicians were provided to the patients by telephone. Such efforts will likely help improving patient safety and reducing delayed treatment.

This study has some limitations to be considered. First, since this study targeted the CRRs generated in musculoskeletal imaging studies read by musculoskeletal attending radiologists, we did not include critical musculoskeletal lesions detected on non-musculoskeletal imaging (e.g., compression fracture detected on chest CT, musculoskeletal active bleeding detected on abdomen CT). Therefore, our study likely substantially underestimates the proportion of all CRRs that are missed fractures. Second, telephonic and direct communications between radiologists and referring physicians were not included for the CRRs owing to problem solving and lack of electronic records. Third, as no detailed manual with specific examples of radiologic CRR is available in our institution, the CRR frequency may have been different depending on the radiologist. Fourth, as radiologists are usually not informed about the clinical outcome of their CRRs, we estimated it from the EMRs. Finally, this study was performed in a single institution with regional trauma center, the incidence of traumatic injury is relatively high. Hence, the results of this study may not reflect the reality of institutions without a trauma center. Nevertheless, the findings allowed us to learn about traumatic lesions that clinicians often miss.

In conclusion, missed fractures were the most common cause of musculoskeletal CRRs. In addition, the ED had the highest prevalence and rate of loss to follow-up action in patients with musculoskeletal CRRs. Physicians in the ED must pay more attention to CRRs, and radiologists should effectively communicate with the referring physicians and provide accurate and timely radiologic reports. A reliable standardized CRR manual is needed, and deployment is necessary across clinical practice.

## Abbreviations

CRR: Critical radiologic report
PACS: Picture Archive and Communication System
EMR: Electronic medical record
ED: Emergency department
